# Study on the relationship and related factors between physical fitness and health behavior of preschool children in southwest China

**DOI:** 10.1186/s12889-024-19269-0

**Published:** 2024-07-02

**Authors:** Ruyun Zou, Kun Wang, Dan Li, Yongsen Liu, Tingran Zhang, Xiudong Wei

**Affiliations:** 1 College of Marxism, Chongqing College of Finance and Economics, Chongqing, 402160 China; 2 College of Physical Education, Research Centre for Exercise Detoxification, Southwest University, Chongqing, 400715 China; 3 Chongqing Science City Bashu Secondary School, Chongqing, 401331 China; 4 Krirk University, Bangkok, 10220 Thailand; 5 College of Physical Education, Chongqing University of Arts and Sciences, Chongqing, 402160 China

**Keywords:** Preschoolers, Physical fitness, Healthy behavior, Physical activity, Screen time

## Abstract

**Objective:**

To investigate the physical fitness level and health behavior status of preschool children in China, explore the relationship between physical fitness and health behavior, and further reveal the main factors affecting health behavior, to provide a reference for improving the physical fitness level of preschool children and maintaining healthy behavior.

**Methods:**

A total of 755 preschool children (394 boys and 361 girls, aged 4.52 ± 1.11 years) were selected from Chongqing and Liupanshui in China by cluster random sampling method for questionnaire survey and physical monitoring, and SPSS21.0 software was used to process and analyze the data.

**Results:**

(1) Heart rate (*p* = 0.015), protein content (*p* < 0.001), and time spent on the balance beam (*p* < 0.001) were significantly lower in boys than in girls, while BMI (*p* = 0.012), muscle mass (*p* < 0.001), and distance of standing long jump (*p* < 0.001) were significantly higher in boys than in girls. Meanwhile, systolic blood pressure (*p* = 0.004) and diastolic blood pressure (*p* = 0.001) of rural children were significantly higher than those of urban children, while BMI (*p* < 0.001) and sitting forward flexion (*p* = 0.019) were significantly lower than those of urban children. (2) The light-intensity physical activity (LPA) and moderate to vigorous physical activity (MVPA) of boys were significantly higher than that of girls (*p* < 0.001), and the MVPA of urban children was significantly higher than that of rural children (*p* = 0.001), and the former participated in sports classes more frequently (*p* < 0.001). (3) There was a significant correlation between physical activity (PA) and physical fitness indicators of preschoolers. Participating in sports interest classes was only significantly correlated with systolic blood pressure (*r* = 0.08) and sitting forward flexion (*r* = 0.09). (4) The PA level of preschool children was related to gender, household registration, kindergarten nature, age, residence environment, parental support, and participation degree. Participation in sports interest classes was related to gender, the nature of the kindergarten, household registration, age, and parent participation. Daily screen time was related to household registration, the nature of the kindergarten, the environment of residence, and the value perception of parents.

**Conclusions:**

There were different degrees of correlation between preschool children’s physical fitness and health behaviors, and children’s health behaviors were closely related to gender, environment, parents, and other factors. Therefore, how to increase the protective factors of children’s health behaviors and controlling the risk factors may be crucial to promoting the development of good health behaviors and improving the physical fitness of preschool children.

## Introduction

Physical fitness represents an individual’s ability to perform physical activities, including muscle strength and endurance, body composition, and flexibility [1]. The age of 3–6 was the key period of individual growth and development, during which physical development entered a period of rapid growth and physical fitness was constantly improved [2]. However, in China, years of physical monitoring data showed that the continuous decline in physical fitness of preschool children has not been effectively improved [3, 4], and with more sedentary lifestyles among children, the rate of obesity and overweight was constantly rising [5]. Globally, childhood overweight and obesity are projected to increase by 60 percentiles, reaching an estimated 250 million children by 2030 [6, 7]. As the foundation period of adolescent physical fitness, preschool children’s physical fitness level was not only closely related to growth and development and adult body health [8, 9] but also related to the future prosperity and development of the nation [10]. Therefore, the scientific monitoring of preschool children’s physical fitness and the clarification of its related factors are undoubtedly a major research focus.

However, the physical fitness of preschool children is a comprehensive variable, which is easily affected by multiple factors. Firstly, the physical fitness level of preschoolers was mainly affected by factors such as heredity [11], parents and family conditions [12], living environment [13], and sleep quality [14]. Secondly, researchers have paid extensive attention to the relationship between preschool children’s health behaviors and their physical fitness, among which health behaviors generally refer to various activities widely involved in daily life to promote physical and mental health and physical fitness, including intake of balanced nutrition, adequate sleep, reduction of screen time, and regular exercise [15]. Studies have shown that health behaviors were closely related to the physical fitness of preschool children, such as physical activity (PA) [16–19], screen time [20, 21], and dietary nutrition [22, 23] have important effects on their physical fitness. Moreover, studies on demographic differences showed that there was significant gender [24, 25] and urban and rural differences in children’s physical fitness [26]. For example, boys have better physical fitness than girls, especially muscle strength, and urban children have better physical shape and flexibility than rural children. These results suggested that there were certain demographic differences in preschool children’s physical fitness, and it was closely related to health behavior, so it may be of great significance to investigate the relationship between their physical fitness and health behavior for the improvement of physical fitness.

Among many health behaviors, PA was considered to be the key to affecting the physical fitness of preschoolers [27, 28]. According to the Exercise Guidelines for Children (3–6 years old) issued by the Chinese government in 2018, preschoolers should spend more than 180 min on various types of physical activities throughout the day, including no less than 60 min of moderate or above intensity physical activities in total [29]. The World Health Organization recommends that young children (5 years and under) [30] and children and adolescents (5 to 17 years) [31] should do an average of at least 60 min of moderate-to-vigorous exercise per day while limiting screen time. A study showed that moderate to vigorous physical activity (MVPA) has a positive influence on improving preschoolers’ physical fitness and replacing light-intensity physical activity (LPA) with MVPA was the best way to increase the physical fitness of preschoolers [32]. Meanwhile, improving the level of PA has positive effects on preschoolers’ mental health [33, 34], cognitive ability [35], and academic performance [36] to varying degrees. In other words, appropriate PA could not only promote the improvement of physical fitness, but also improve psychological, cognitive, and academic performance [37]. The preschool period was the best time for children to learn and improve their motor skills, and a positive physical and health education experience was also easy to form during this period [38] ensuring the necessary amount of PA was essential for healthy growth of preschoolers.

Screen time was another important factor affecting the physical fitness of preschoolers. Excessive daily screen time has been linked primarily to rising rates of obesity among preschoolers. For example, studies have shown that a poor lifestyle (such as screen time) was associated with an increased rate of obesity in preschool children [39], and excessive daily screen time was a risk factor for increasing abdominal obesity and BMI in preschool children [24, 40]. Therefore, maintaining an appropriate amount of physical activity while controlling behaviors such as screen time may be an effective way to improve or improve the physical fitness of preschool children. However, due to the young age of preschool children, their physical activity, screen time, and other behaviors are usually closely related to their parents, teachers, and external environment in addition to their limited cognition. Based on this, under the background of the fifth national physique monitoring, this study adopts the method of cluster random sampling to investigate the current situation of physical fitness and health behavior of preschoolers in Chongqing and the Liupanshui City of China and their relationship, and further explore the main influencing factors of health behaviors such as physical activity level, participation in sports interest classes, and daily screen time of preschool children, thus to provide a reference for scientifically leading preschoolers to form healthy behaviors and improve their physical fitness.

## Methods

### Participants

A total of 8 kindergartens in Chongqing and Liupanshui City were randomly selected for testing by cluster random sampling method (These two cities are designated test sites for China’s national physical fitness test in Southwest China). Meanwhile, 80 to 120 preschool children were randomly selected from each kindergarten in a ratio of about 1:4. A total of 800 preschoolers aged 3–6 years old were recruited, and 755 children completed the physical fitness test with complete and valid data (Table 1). Among them, 394 were boys, with an average body height of 1.08 ± 0.07 m, body mass of 18.75 ± 3.21 kg, and age of 4.67 ± 1.12 years. There were 361 girls with an average body height of 1.06 ± 0.08 m, body mass of 17.80 ± 3.02 kg, and age of 4.36 ± 1.08 years.

**Table 1:** Basic information and index assignments of participants (*n* = 755)

Variable	Category and Assignment	*n*	Variable	Category and Assignment	*n*
Gender	Boys = 1	394	Caregiver	Parents = 1	488
	Girls = 2	361		Other = 2	267
Household registration	Rural = 1	326	Residence with outdoor sports facilities	Yes = 1	467
	Urban = 2	429		No = 2	288
Years	Three years = 1	168	Father’s educational background	Junior high school and below = 1	82
	Four years = 2	226		High school = 2	305
	Five years = 3	158		University and above = 3	368
	Six years = 4	203	Mother’s educational background	Junior high school and below = 1	100
Kindergarten Nature	Public = 1	351		High school = 2	292
	Private = 2	404		University and above = 3	363
PA	Low activity amount = 0	295	Participation in sports interest classes	No = 0	440
	Moderate to high activity amount = 1	460		Yes = 1	315
			Daily screen time	≥ 1 h = 0	367
				<1 h = 1	388

*Note* The small number in “Category and assignment” means that it is taken as the base ratio in logistics regression, such as “1” in “gender” or “0” in " PA amount “

Participants’ inclusion criteria: (1) Preschoolers aged 3 to 6 years; (2) All four limbs, no disability; (3) Parents/guardians agree and sign the informed consent form. Exclusion criteria: (1) A history of cardiovascular and respiratory diseases; (2) After assessment with the Physical Activity Readiness Questionnaire (PAR-Q) [41] (to be completed by a parent or guardian), participants can participate in moderate-intensity physical activities or above. This study was approved by the Ethics Committee of the School of Physical Education, Southwest University (SWU-TY202105), and followed the Declaration of Helsinki. Written informed consent was obtained from all participants, moreover, because of the young age of the participants, we also obtained the approval of their parents, who signed the parental informed consent.

### Questionnaire survey

#### Process

During the physical fitness test, the parents or guardians of preschoolers were surveyed by the questionnaire survey. The Fifth National Physical Fitness Monitoring Questionnaire (3 to 6-year-old children) [42] developed by the General Administration of Sport of China was adopted. It was jointly developed by the Chinese government and the General Administration of Sport of China and was an authoritative questionnaire for national physical fitness monitoring. To ensure the authenticity of the questionnaire filling, we gathered the parents or guardians of preschoolers who participated in the survey in different periods respectively to the designated test site and fully explained the questionnaire content and precautions to them before handing out the questionnaire. Moreover, questionnaires were distributed and collected on-site, and participants (parents or guardians) were asked to recall and fill out the questionnaire based on their own children’s real situation, and each questionnaire took about 20 min to complete. A total of 800 questionnaires were sent out in this study, and 755 were effectively received, with an effective recovery rate of 94.38 percentiles. The contents of the questionnaire mainly include demographic information (such as gender and age), family structure (such as both parents are together or single single-parent families), environment of residence (such as city or country, whether there are outdoor sports facilities in residence), educational level of parents, physical activity amount, daily screen time, daily physical activities (such as running, fun games, and sports training classes), and parents’ support for their children’s sports. It should be pointed out that according to the framework of the questionnaire in this study, the contents related to the health behaviors of preschool children mainly involve daily screen time, amount of physical activity, and participation in sports interest training courses. Therefore, these three variables were used in this study to reflect the health behaviors of preschool children.

#### Reliability and validity of the questionnaire

To test the reliability and validity of the questionnaire, the questionnaire was scored by six infant health experts before the formal investigation, with an average score of 97.18 points. Meanwhile, the retest method was used to test the reliability of the questionnaire, and the parents or guardians of 68 preschoolers in two of the kindergartens were randomly selected to repeat the survey, and the interval between the two tests was 10 days. The intra-group correlation coefficient (ICC) was used to test, and the retest reliability was 0.85. It shows that the whole questionnaire has good reliability and measurement validity. Among them, the reliability and validity of the Parents Sport Support Scale and Physical Activity Rating Scale were as follows:

The Parents Sport Support Scale was used to evaluate the parental exercise support of preschoolers, which was assessed by 9 items. The Likert 5-point scale was adopted, according to the option “strongly disagree-strongly agree”, it was scored as 1 to 5 points respectively, the higher the score, the more positive the attitude. A total of 3 common factors were extracted through factor analysis, including value perception (4 items, such as “Do you think your child’s physical fitness can be enhanced by playing sports games”), sports support (3 items, such as “Do you encourage your child to go out for sports games in your spare time”), participation in accompaniment (2 items, such as “Would you like to arrange regular sports games with your child?“), after direct oblique rotation, the progressive contribution rate of the three common factors reaches 61.72 percentiles. After the internal consistency test, the Cronbach α of value perception was 0.86, the Cronbach α of sports support was 0.84, and the Cronbach α of participation escort was 0.87. The measurement model validation results: x^2^/df = 1.91, RMSEA = 0.04, AGFI = 0.98, TLI = 0.97, CFI = 0.99, IFI = 0.97, GFI = 0.97. It shows that the questionnaire as a whole has good reliability and measurement validity.

The Physical Activity Rating Scale (PARS-3) was adopted to assess the PA amount of preschoolers [43], which includes three dimensions PA intensity, activity time, and activity frequency. The Likert 5-point scale was adopted, and the PA amount = activity intensity × (activity time − 1) × activity frequency, the score range was 0 to 100 points, and the assessment criteria for PA amount were: low PA amount ≤ 19 points, moderate PA amount range was 20 to 42 points, high PA amount ≥ 43 points. The retest reliability of this scale is high, and the correlation coefficient *r* = 0.82. It should be pointed out that preschoolers’ PA consists of two parts school physical activity and weekend PA, so the scale was filled by teachers and parents respectively according to the actual situation of children in different scenarios.

### General fitness test

The general fitness testing of preschoolers was conducted in the sampled kindergartens by qualified professionals trained by the National Physical Fitness Monitoring Center, and the testing time was 8:30 to noon and 14:00 to 17:30. The testing equipment adopts the equipment designated by the national physical fitness monitoring, and conducts general fitness testing and comprehensive rating according to the test methods and scoring standards of the “National Physical Fitness Monitoring Work Manual (Children Part)”. The test indicators were divided into three parts: cardiovascular test, body composition analysis, and general fitness test.

#### Cardiovascular test

Resting heart rate and blood pressure. The resting heart rate, systolic blood pressure (SBP), and diastolic blood pressure (DBP) were measured using an Omron HBP9020 automatic electronic blood pressure monitor (Omron, Osaka, Japan). The measurement method was to measure the blood pressure of the right upper arm after sitting for 5 min. Specifically, the armband roll was tied 2–3 cm above the elbow joint of the upper arm, and the armband was at the same level as the heart. The armband was automatically pressurized and measured according to the starting key, and the measurement should be kept quiet during the pressure measurement. The measurement process takes about 50 s. It should be pointed out that to improve the accuracy of the data, the measurement was repeated once at an interval of two min, and the average value of the two counts was recorded.

#### Body composition analysis

The body composition data of preschoolers were measured by InBody (InBody-270, Korea), which included body fat percentage (BFP), waist-to-hip ratio (WHR), muscle mass (MM), and protein. During the test, participants should stand barefoot on the instrument as required, and hold the electrode pads of the handle with both hands, and the test duration was about the 50s. It should be pointed out that the WHR was the measurement of the waist and hip circumference using a tape measure, and then input the corresponding values into the computer InBody software to calculate the WHR. In addition, to examine the body mass index (BMI) of preschoolers, their body height and weight were measured and calculated according to the formula “Body Mass Index (BMI) = weight/(height * height)”, the unit of weight is kg, and the unit of height is m.

#### General fitness test

The general fitness test includes a total of six test items, as follows:

(I) Grip strength (GS): A hand dynamometer is an evaluation tool that’s used to measure isometric grip force (hand grip strength) (Xinman WCS-100 model, Shanghai, China). During the test, participants need to hold their hands firmly. At this time, the data on the LCD screen starts to refresh and the measurement data can be read until there are no more new measurement peaks, records were made in kg units.
(II) Sitting forward flexion (SFF): The electronic sitting forward flexion tester (Lingkang LK-T5016, Jiangsu, China) is an assessment tool used to measure the possible range of motion of the torso, waist, buttocks, and other joints at rest, mainly reflecting the ductility and flexibility of the joints, ligaments, and muscles in these parts. During the test, participants should extend their legs straight, keep their heels together, and separate their toes naturally. Then, palms down, arms together and extended flat, upper body flexion forward, and two fingers moving forward at a constant speed until they cannot push the touchpad, were recorded in centimeters (cm).
(III) Standing long jump (SLJ): A standing long jump tester is an assessment tool for measuring lower limb explosive power (Hengkang HK-6000-TY, Shenzhen, China). After pre-swing, both feet will take off in place at the same time without pad or tandem jumping, and the instrument will automatically measure the vertical distance between the back edge of the take-off line and the back edge of the nearest landing site, which was recorded in centimeters (cm).
(IV) Continuous jump of both feet (CJBF): A two-foot continuous jump tester is an assessment tool used to measure lower limb jumps and coordination (BESTWEN, 7-inch portable, Jiangsu, China). After hearing the instruction to start, participants should jump forward with their feet together. After the first foot lands on the mat, perform a second jump in a row, and the instrument will automatically record the distance between the two jumps, which was recorded in meters (m).
(V) Balance beam (BB): The balance beam test instrument is an assessment tool used to measure balance ability (Boffei, Zhejiang, China). Participants should stand behind the “starting line”, face the balance beam, and raise their arms horizontally. After hearing the command “start”, their feet should move forward to the “finish line” alternately. When either foot exceeds the “finish line”, the instrument will automatically record the time, which was recorded in seconds (s).
(VI) 15 m obstacle run (15 m OR): The obstacle run test instrument is an assessment tool for a comprehensive measurement of reflexes, coordination, and speed (LK-T6013, Jiangsu, China). When participants heard the start instruction, they quickly circumnavigated the six obstacles, and the instrument automatically recorded the time when they reached the finish line, which was recorded in seconds (s).

It should be pointed out that the subjects do not need to conduct targeted warm-up activities before each test, and professional testers will give technical explanations before each test, practice once or twice during the test, and then conduct formal tests. Each group was tested in the same order, starting with strength, speed/agility, strength, endurance, and ending with flexibility.

### Statistical analysis

All data were processed and analyzed using SPSS21.0 software. The independent sample t-test (also known as the student’s t-test) was used to analyze the status quo of physical fitness and health behaviors of preschoolers, the Pearson correlation analysis was used to investigate the relationship between physical fitness and health behaviors, and the logistic regression analysis was used to investigate the influencing factors of preschoolers’ health behaviors. The significance level of all indexes was set at *p* < 0.05.

## Results

### The physical fitness status of preschoolers

The survey results of the physical fitness status of preschoolers showed (Table 2): (1) In terms of cardiovascular test, there were no significant gender differences in SBP (*p* = 0.619) and DBP (*p* = 0.160) in preschoolers, but the HR of boys was significantly lower than that of girls (*p* = 0.015). Meanwhile, the SBP (*p* = 0.004) and DBP (*p* = 0.001) of rural children were significantly higher than those of urban children, but there was no significant difference in HR between urban and rural areas (*p* = 0.136). (2) In terms of body composition analysis, the BMI (*p* = 0.012) and MM (*p* < 0.001) of boys were significantly higher than those of girls, while the protein content of girls was significantly higher than that of boys (*p* < 0.001), and the rest of the indicators showed no significant gender differences. Meanwhile, the BMI of urban children was significantly higher than that of rural children (*p* < 0.001), and there was no significant difference between urban and rural areas in other indicators. (3) In terms of physical fitness, boys were significantly better than girls in SLJ (*p* < 0.001), BB (*p* < 0.001), and GS (*p* < 0.001), while there was no significant gender difference in other indicators. The SFF of urban children was significantly better than that of rural children (*p* = 0.019), and there was no significant difference between urban and rural areas in other indicators.

**Table 2:** Demographic characteristics of physical fitness of preschoolers (*n* = 755)

Category	Index	Ranges of variation	Boys (394)	Girls (361)	*p*	Rural (326)	Urban(429)	*p*
General fitness test	SFF (cm)	(2.10–21.80)	8.70 ± 5.48	8.19 ± 5.25	0.193	7.92 ± 5.92	8.86 ± 4.88	0.019
	SLJ (cm)	(35.00-135.00)	75.38 ± 25.68	67.47 ± 24.16	< 0.001	70.57 ± 24.73	72.38 ± 25.65	0.331
	CJBF (s)	(2.40–5.60)	8.11 ± 4.04	8.64 ± 4.45	0.086	8.39 ± 4.50	8.34 ± 4.05	0.874
	BB (s)	(4.80–29.60)	10.54 ± 6.54	12.70 ± 9.39	< 0.001	11.36 ± 8.81	11.74 ± 7.53	0.530
	15 m OR (s)	(5.60–15.40)	8.28 ± 1.86	8.53 ± 1.84	0.069	8.42 ± 1.59	8.39 ± 2.03	0.809
	GS (kg)	(2.70–9.10)	5.44 ± 2.62	4.48 ± 2.18	< 0.001	4.88 ± 2.54	5.05 ± 2.41	0.353
Body composition analysis	BMI	(7.73–22.97)	15.96 ± 1.51	15.67 ± 1.60	0.012	15.55 ± 1.68	16.02 ± 1.43	< 0.001
	BFP (percentiles)	(9.00-33.80)	15.41 ± 2.26	15.49 ± 2.54	0.676	15.63 ± 2.66	15.30 ± 2.16	0.069
	WHR	(0.10–0.80)	0.45 ± 0.11	0.44 ± 0.10	0.058	0.44 ± 0.10	0.45 ± 0.11	0.112
	MM (kg)	(7.40–27.20)	13.47 ± 2.37	12.12 ± 2.32	< 0.001	12.89 ± 2.86	12.78 ± 2.06	0.543
	Protein (kg)	(1.90–6.40)	3.03 ± 0.59	3.41 ± 0.74	< 0.001	3.25 ± 0.81	3.18 ± 0.59	0.175
Cardiovascular test	HR (beat)	(59.00-134.00)	92.12 ± 11.29	94.23 ± 12.44	0.015	93.87 ± 12.33	92.57 ± 11.53	0.136
	SBP (mmHg)	(84.00-109.00)	95.12 ± 6.35	95.35 ± 6.17	0.619	95.98 ± 6.38	94.66 ± 6.11	0. 004
	DBP (mmHg)	(57.00–80.00)	66.55 ± 5.54	67.10 ± 5.30	0.160	67.57 ± 5.51	66.24 ± 5.31	0.001

*Note* “SFF” means “Sitting forward flexion”, “SLJ” means “Standing long jump”, “CJBF” means “Continuous jump of both feet”, “BB” means “Balance beam”, “15m OR” means “15m Obstacle Run”, “GS” means “Grip strength”, “BMI” means “Body mass index”, “BFP” means “Body fat percentage”, “WHR” means “Waist to hip ratio”, “MM” means “Muscle mass”, “HR” means “Heart rate”, “SBP” means “Systolic blood pressure”, “DBP” means “Diastolic blood pressure”

### Status of health behaviors of preschoolers

The results of the status quo of health behaviors of preschoolers showed (Table 3): (1) In terms of PA, among the low-activity groups, the activity level of boys was significantly higher than that of girls (*p* < 0.001), while there was no significant difference between rural and urban children (*p* = 0.189). In the MVPA group, the activity level of boys was significantly higher than that of girls (*p* < 0.001), and the activity level of urban children was significantly higher than that of rural children (*p* = 0.001). It should be pointed out that among the surveyed preschoolers, very few people have reached a high level of PA, so this study combined moderate and high PA into one category according to the actual situation, that is, MVPA. (2) In terms of participation in sports interest classes, there was no significant gender difference (*p* = 0.176), but the participation of urban children was significantly higher than that of rural children (*p* < 0.001). (3) There were no significant gender (*p* = 0.057) and urban-rural (*p* = 0.693) differences in daily screen time.

**Table 3:** Demographic characteristics of health behaviors of preschoolers (*n* = 755)

Category	Index	Boys	Girls	*p*	Rural	Urban	*p*
PA amount	Low activity amount	14.78 ± 3.87	9.95 ± 6.42	< 0.001	11.34 ± 6.66	12.27 ± 5.36	0.189
	MVPA	36.70 ± 12.78	29.33 ± 5.07	< 0.001	31.77 ± 7.55	35.14 ± 12.67	0.001
Participate in sports interest classes	Frequency	1.13 ± 1.51	0.99 ± 1.39	0.176	0.80 ± 1.35	1.26 ± 1.50	< 0.001
Daily screen time	Hour	1.17 ± 0.97	1.31 ± 1.07	0.057	1.26 ± 0.99	1.23 ± 1.04	0.693

*Note* Low activity amount ≤ 19 points, 19 points < MVPA. MVPA: moderate to vigorous physical activity

### The relationship between physical fitness and healthy behavior of preschoolers

The correlation analysis showed (Table 4): There was a close relationship between physical fitness and healthy behaviors of preschoolers. (1) There was a significant correlation between physical fitness and PA, which was manifested as a significant negative correlation between PA amount and HR (*r*=-0.19), SBP (*r*=-0.08), and DBP (*r*=-0.12). The PA amount was significantly positively correlated with BMI (*r* = 0.12), WHR (*r* = 0.18), MM (*r* = 0.15), and protein (*r* = 0.09), and was significantly negatively correlated with BFP (*r*=-0.11). The PA amount was significantly positively correlated with SFF (*r* = 0.09), SLJ (*r* = 0.19), and GS (*r* = 0.16), while it was significantly negatively correlated with CJBF (*r*= -0.20), BB (*r*=-0.35), and the 15 m OR (*r* = -0.15). (2) In terms of sports interest classes, weekly participation in sports interest classes was only significantly positively correlated with SBP (*r* = 0.08) and SFF (*r* = 0.09), but had no significant correlation with other physical fitness indicators (3) In terms of screen time, there was no significant direct correlation between daily screen time and physical fitness of preschoolers.

**Table 4:** Correlation between physical fitness and healthy behaviors (*n* = 755)

	HR	SBP	DBP	BMI	BFP	WHR	MM	Protein	SFF	SLJ	CJBF	BB	15 m OR	GS
M1	-0.19	-0.08	-0.12	0.12	-0.11	0.18	0.15	0.09	0.09	0.19	-0.20	-0.35	-0.15	0.16
p	< 0.001	0.024	0.001	0.001	0.003	< 0.001	< 0.001	0.010	0.012	< 0.001	< 0.001	< 0.001	< 0.001	< 0.001
M2	0.02	0.08	0.06	0.04	-0.04	0.03	-0.01	0.03	0.09	0.01	-0.06	-0.04	-0.05	-0.02
p	0.664	0.040	0.104	0.313	0.231	0.421	0.917	0.426	0.012	0.999	0.079	0.321	0.190	0.652
M3	-0.03	0.03	0.04	0.02	0.06	-0.04	-0.01	0.00	-0.04	0.01	0.02	0.07	0.01	-0.01
p	0.363	0.455	0.232	0.621	0.098	0.245	0.959	0.952	0.342	0.996	0.572	0.054	0.820	0.730
Mean	93.13	95.23	66.81	15.82	15.44	0.44	12.82	3.21	8.46	71.60	8.36	11.58	8.40	4.98
SD	11.89	6.26	5.43	1.56	2.39	0.10	2.44	0.70	5.37	25.26	4.25	8.10	1.85	2.47

*Note* “M1” refers to “weekly PA”, “M2” refers to “weekly participation in sports interest classes”, and “M3” refers to “daily screen time”

### Influencing factors of preschoolers’ health behavior

#### Construction of binary logistic regression model

The logistic regression analysis theoretically does not require that each index variable must obey the conditions of linearity, independence, and normality. Therefore, this study takes the influencing factors of preschoolers’ health behavior as independent variables and selects three main aspects of children’s health behavior, that is PA, participation in sports interest classes, and screen time as outcome variables to establish regression models respectively (Model 1, Model 2, Model 3, respectively), to further explore the influence of influencing factors on different health behaviors of preschoolers, and the Odds Ratio (OR) was introduced into the regression model [44, 45]. Usually, OR > 1 indicates that the factor was a favorable factor, OR < 1 indicates that the factor was an unfavorable factor, and OR = 1 indicates that the factor has nothing to do with the outcome variable.

#### Test of logistic regression model and odds ratio analysis

From the global test results of the model, the chi-square value and significance level of the likelihood ratio (Likelihood Ratio) corresponding to the model reaches the ideal level, model 1 (x^2^ = 169.19, *p* < 0.001), model 2 (x^2^ = 121.16, *p* < 0.001), model 3 (x^2^ = 48.57, *p* < 0.001), indicating that all three models were statistically significant. From the model’s goodness-of-fit test results, model 1 (x^2^ = 841.11, *p* > 0.075), model 2 (x^2^ = 904.70, *p* = 0.112), model 3 (x^2^ = 997.98, *p* = 0.241), the corresponding probability p values of the three models were all greater than 0.05, which further confirms that the fitting effect of the model was acceptable.

From the odds ratio correlation analysis of Model 1, we could see that the PA of preschoolers was mainly related to the following six factors: (1) From the perspective of gender, the odds ratio of “boys/girls” Exp(b) = 1.413 (*p* = 0.031), indicating that the proportion of boys doing MVPA was 1.413 times that of girls, that is, the former was significantly higher than the latter. (2) From the perspective of household registration, the advantage ratio of “rural/urban” was Exp(b) = 0.405 (*p* = 0.012), indicating that the MVPA performed by rural children was 0.405 times that of urban children, that is, the former was significantly lower than the latter. (3) From the perspective of the nature of kindergartens, the advantage ratio of “public/private” was Exp(b) = 2.280 (*p* = 0.019), indicating that the MVPA performed by children in public kindergartens was 2.280 times that of children in private kindergartens, that is, the former was significantly higher than the latter. (4) In terms of age, the odds ratio Exp(b) of “3 years old/6 years old” was Exp(b) = 0.573 (*p* = 0.027), and the odds ratio Exp(b) of “4 years old/6 years old” was Exp(b) = 0.627 (*p* = 0.045), indicating that 3-year-old and 4-year-old children’s MVPA were 0.573 and 0.627 times that of 6-year-old children, respectively, that is, 6-year-old children’s PA was higher. (5) From the perspective of the outdoor sports facilities in the place of residence, the odds ratio of “yes/no” Exp(b) = 1.455 (*p* = 0.043), indicating that the MVPA performed by children with sports facilities in their residence was 1.455 times that without the corresponding facilities, that is, the former was significantly higher than the latter. (6) From the perspective of parental sports support, the odds ratio Exp(b) of “sports support” was Exp(b) = 1.138 (*p* = 0.039), and the odds ratios Exp(b) of “participation accompanying” was Exp(b) = 1.112 (*p* = 0.021), indicating that for each unit of increase in these two factors, the proportion of preschoolers participating in MVPA will increase to 1.138 and 1.112 times, respectively.

From the correlation analysis of the odds ratio of Model 2, we could see that whether preschoolers participate in sports interest classes was mainly related to the following five factors: (1) In terms of gender, the odds ratio of “boys/girls” Exp(b) = 0.675 (*p* = 0.022), indicating that the proportion of boys participating in sports interest classes was only 0.675 times that of girls, that is, the former was significantly lower than the latter. (2) From the perspective of household registration, the advantage ratio of “rural/urban” was Exp(b) = 0.341 (*p* < 0.001), indicating that rural children’s participation in sports interest classes was 0.341 times that of urban children, that is, the former was significantly lower than the latter. (3) In terms of the nature of kindergartens, the advantage ratio of “public/private” was Exp(b) = 1.848 (*p* = 0.035), indicating that children in public kindergartens participate in sports interest classes 1.848 times that of children in private kindergartens, that is, the former was significantly higher than the latter. (4) From the perspective of age, the advantage ratio of “3 years old/6 years old” was Exp(b) = 0.369 (*p* < 0.001), indicating that 3-year-old children participating in sports interest classes were 0.369 times that of 6-year-old children, that is, the former was significantly lower than the latter. (5) From the perspective of parental sports support, the odds ratio Exp(b) = 1.192 (*p* < 0.001) of “participation and companionship”, indicating that every time the factor increases by one unit, the proportion of children participating in sports interest classes will increase to 1.192 times.

From the odds ratio correlation analysis of Model 3, we could see that the daily screen time of preschoolers was mainly related to the following four factors: (1) From the perspective of household registration, the advantage ratio Exp(b) of “rural/urban” = 0.573 (*p* = 0.043), indicating that the proportion of rural children’s daily screen time less than 1 h was 0.573 times that of urban children, that is, the former has more screen time per day. (2) From the perspective of the nature of kindergartens, the advantage ratio of “public/private” was Exp(b) = 1.825 (*p* = 0.026), which showed that the daily screen time of children studying in public kindergartens was less than 1 h was 1.825 times that of children in private kindergartens, that is, the former has less screen time per day. (3) In terms of whether there were outdoor sports facilities in the place of residence, the odds ratio of “yes/no” was Exp(b) = 1.416 (*p* = 0.037), indicating that the daily screen time of children with sports venue facilities in their residence was less than 1 h was 1.416 times that of children without corresponding venue facilities, that is, the former has less screen time per day. (4) From the perspective of parental exercise support, the odds ratio was Exp(b) = 1.075 (*p* = 0.011) of “value perception”, indicating that for each unit increase of this factor, the proportion of children’s daily screen time less than 1 h will increase to 1.075 times the original (please see Table 5).

**Table 5:** Statistics on the correlation between health behaviors and constraints of preschoolers

Variables	Model 1: MVPA/Low			Model 2: Participate/Not Participate			Model 3: <1 h/≥1 h		
	Exp(b)	95%CI	*p*	Exp(b)	95%CI	*p*	Exp(b)	95%CI	*p*
X1: Boy/Girl	1.413	(1.007,1.983)	0.031	0.675	(0.482,0.945)	0.022	0.868	(0.633,1.190)	0.379
X2: Rural/Urban	0.405	(0.200,0.821)	0.012	0.341	(0.191,0.610)	0.000	0.573	(0.333,0.984)	0.043
X3: Public/Private	2.280	(1.145,4.538)	0.019	1.848	(1.046,3.267)	0.035	1.825	(1.074,3.102)	0.026
X4: 3-year-old /6-year-old	0.573	(0.349,0.939)	0.027	0.369	(0.225,0.605)	0.000	1.211	(0.772,1.902)	0.404
4-year-old /6-year-old	0.627	(0.398,0.990)	0.045	0.760	(0.494,1.172)	0.214	0.883	(0.586,1.333)	0.554
5-year-old /6-year-old	1.131	(0.682,1.874)	0.633	0.936	(0.594,1.474)	0.775	1.143	(0.737,1.772)	0.550
X5: Parents/Others	0.856	(0.591,1.242)	0.413	1.223	(0.855,1.749)	0.270	0.835	(0.597,1.166)	0.289
X6: Junior high school and below/University and above	0.796	(0.355,1.783)	0.579	0.733	(0.332,1.614)	0.440	1.103	(0.533,2.285)	0.791
High school degree/ University and above	1.061	(0.626,1.798)	0.827	0.863	(0.525,1.419)	0.562	1.054	(0.658,1.688)	0.827
X7: Junior high school and below/University and above	1.515	(0.703,3.264)	0.289	0.526	(0.252,1.099)	0.088	1.780	(0.893,3.548)	0.101
High school degree/ University and above	1.090	(0.653,1.818)	0.742	0.628	(0.386,1.021)	0.061	0.874	(0.553,1.381)	0.563
X8: Yes/No	1.455	(1.012,2.090)	0.043	0.858	(0.604,1.220)	0.394	1.416	(1.021,1.964)	0.037
X9: Value perception	1.052	(0.984,1.124)	0.135	1.022	(0.959,1.089)	0.499	1.075	(1.017,1.137)	0.011
Sports support	1.138	(1.007,1.286)	0.039	0.929	(0.828,1.042)	0.210	1.026	(0.947,1.113)	0.524
Participation accompanying	1.112	(1.016,1.217)	0.021	1.192	(1.089,1.303)	0.000	1.010	(0.938,1.089)	0.784

*Note* Exp(b) represents the odds ratio (OR), which is the base ratio (for example, “female” in “male/female” is the base ratio). Model 1 refers to the weekly PA of preschoolers, Model 2 refers to weekly participation in sports interest classes, and Model 3 refers to daily screen time (such as mobile phones, and TVs). X1 indicates the gender of the child, X2 indicates the household registration, X3 indicates the nature of the kindergarten, X4 indicates the age, X5 indicates the primary caregiver of the child, X6 indicates the educational background of the child’s father, X7 indicates the educational background of the child’s mother, X8 indicates whether the child’s residence has outdoor sports facilities and venues, and X9 indicates the parent’s sports support

## Discussions

### Difference analysis of physical fitness and healthy behavior of preschoolers

In this study, we found that boys’ physical fitness (such as standing long jump, grip strength, balance, et al.) and body composition analysis (such as BMI, muscle mass, et al.) were significantly better than girls’, while girls’ resting heart rate and body protein content were higher high. This was consistent with previous research results, indicating that boys have relatively good physical conditions [46, 47], the physiological structural characteristics of boys tend to make them have better muscle strength, while girls were more likely to have qualities such as greater flexibility [25]. However, this study did not find that girls had better performance in sitting forward flexion, which may be related to the fact that the flexibility of preschoolers was still in the initial stage of formation. In addition, the rural children had higher systolic blood pressure and diastolic blood pressure, lower BMI, and sitting forward flexion. This was similar to the study of Zhang et al. [26], which showed that urban young children have relatively better cardiovascular system development and better body composition analysis and flexibility. Overall, there were certain demographic differences in the physical fitness of preschoolers, and we speculate that this may be related to their genetics, living environment, and lifestyle factors.

Meanwhile, we found that boys were generally more physically active than girls. Studies have pointed out that the PA level of children is closely related to their growth and development, especially the MVPA [48]. In general, boys were more active than girls [49], and it was easier for them to do more physical activities in preschool. The study has shown that boys generally show a greater MVPA than girls [50], which may be one reason why boys have better physical fitness. A recent large sample study also found that preschool boys had significantly higher daily MVPA than girls [51] and that boys participated in most different categories of MVPA on weekdays and weekends than girls [52]. In addition, compared with rural children, urban children have significantly higher levels of middle and high activity, and the latter participate in sports classes significantly more frequently than the latter. Research showed that parents of urban children were better than parents of rural children in terms of education level, economic income, and educational concept [26], so they were more willing to let their children participate in sports classes, such as dancing, football, and swimming, which was one of the potential factors for urban children to have higher PA. Overall, this study identified gender and urban-rural differences in physical fitness and physical activity of preschool children in Southwest China, which was conducive to the formulation of follow-up personalized health promotion programs.

### The relationship between physical fitness and healthy behaviors of preschoolers

This study found a significant correlation between physical fitness and PA in preschoolers, as higher levels of PA were associated with lower heart rate and blood pressure, and better body composition analysis and fitness in preschoolers. There was a close relationship between physical fitness and PA in children [20, 53], especially MVPA, which was believed to be effective in improving muscle strength, explosiveness, balance, agility, and aerobic fitness in preschoolers [54]. Meanwhile, MVPA has a positive influence on the improvement of preschool children’s body composition and physical fitness [55], and this study further confirmed the linear relationship between physical activity and children’s body composition, physical function, and physical fitness, increasing MVPA and reducing low-intensity PA was one of the effective ways to improve the physical fitness of preschoolers. However, the correlation between participation in sports interest classes and the physical fitness of preschoolers was weak and only correlated with systolic blood pressure and sitting forward flexion. This result may be related to two factors. One was the type of sports hobby classes involved, for example, some hobby classes pay more attention to cultivating children’s motor skills rather than physical fitness. Second due to preschoolers being too young, they tend to do low-intensity physical activities in sports classes, which cannot effectively improve their general fitness test. Moreover, Wang et al. [20] found that controlling the screen time of preschoolers was a protective factor for their flexibility, but this study did not find a significant direct correlation between daily screen time and the physical fitness of preschoolers. Research suggests that this may be related to participation in sports interest classes [20], therefore, it was speculated that screen time does not directly affect the physical fitness of young children but indirectly affects their physical fitness level by reducing healthy behaviors such as PA [56]. It can be seen that maintaining a moderate amount of physical activity (especially MVPA) can directly or indirectly promote the physical fitness of preschool children, and actively participating in sports classes and reducing screen time are protective factors for physical fitness.

### Analysis of influencing factors on preschoolers’ healthy behavior

Given the important influence of health behavior on the physical fitness of preschoolers, this study used logistic regression analysis to deeply explore the influencing factors of preschoolers’ health behavior. The results showed that the PA of preschoolers was mainly related to many factors, such as gender, household registration, the nature of kindergarten, age, living environment, and parental exercise support. Hinkley et al. [57] identified the relevant factors affecting the PA of preschoolers from different domains, including children’s gender, outdoor time, and parents’ PA levels, based on the socioecological model. Tucker [58] pointed out that boys were more active than girls, and that the availability of facilities, play space, and encouragement from parents or kindergarten teachers played an important role in the PA of young children. Some studies have also pointed out that social and cultural factors such as spatial environment and family roles, physical activities of parents, and encouragement from kindergarten teachers have important influences on children’s physical activities, of which environmental factors have a greater impact [59]. Meanwhile, some researchers have conducted preliminary research on the influencing factors of children’s 24-hour PA behavior [60]. Among them, two survey studies on infants and young children (3.6 months to 4.2 years old) in Australia and Belgium found that there was no significant gender difference in the 24-hour PA of infants and young children [61, 62]. Kracht et al. [63]. explored the influence of age, race, and family economic level on the 24-hour activity compliance of children aged 3–4 in the United States, and found that the influence of race and family economic level reached a significant level, while age was not a significant difference. It can be seen that the factors affecting the amount of PA in preschoolers involve a wide range [21, 64], this study further revealed the proportion of influence of gender, household registration, kindergarten nature, age, residence environment, and parental exercise support on their physical activity level through logistic regression analysis. It should be noted that given the differences in different studies, we speculate that it may be related to the research paradigm and the demographic differences of the respondents, the interaction relationship and mechanism among the factors still need to be further explored.

In terms of the influencing factors of participation in sports interest classes, this study finds that boys were less likely to participate in sports interest classes, which could be explained by the fact that boys were lively and had more leisure activities, so parents were more inclined to let them participate in art interest classes such as painting and music and tend to let girls participate in interest classes such as dance. As for children with urban household registration, the frequency of participating in sports interest classes was higher, research suggests that it may be related to the economic level of the family and the educational concept of parents [63, 65]. Meanwhile, compared with private kindergartens, children in public kindergartens participate in sports interest classes more frequently, which may be related to the school’s curriculum content and teachers’ teaching methods. Compared with 6-year-old children, the participation frequency of 3-year-old children was lower. This may be because 3-year-old children were too young, their physiological functions were not fully developed, and they were not suitable for participating in too many sports interest classes, many training institutions also have clear age restrictions. In addition, the degree of parental participation and companionship also affects the frequency of children’s sports interest classes, the higher the degree of companionship, the higher the frequency of children’s participation. It should be noted that previous studies paid little attention to the influencing factors of preschool children’s participation in sports interest classes, but this study clarified the proportion of gender, household registration, kindergarten nature, age, and parents’ sports support on the participation of sports interest classes, which has reference value for investigating the characteristics of children’s participation in sports interest classes.

In terms of daily screen time, studies have shown that excessive screen time in infants and young children not only leads to decreased vision and sleep quality, and increased overweight and obesity [66, 67], but also impairs cognitive function in young children [68]. The findings showed that rural children spend more screen time per day than urban children, this may be because the parents of rural children work outside all year round, the supervision of their supervisors (such as grandparents) was relatively loose, and rural children have relatively few leisure and entertainment activities, resulting in increased screen time. The screen time of children in public kindergartens was significantly lower than that in private kindergartens, which may be related to the educational methods of schools of different natures. Meanwhile, children who live in abundant outdoor sports facilities and venues have relatively less screen time per day, indicating that the environment has a certain influence on children’s behavior, and a good sports environment could promote the corresponding sports behavior of young children, thereby reducing screen time. In addition, the higher the parents’ perception of the value of children’s sports participation behavior, the lower the children’s daily screen time, again indicating that parents’ perceptions could directly or indirectly affect children’s behavior. Overall, excessive screen time was likely to harm the physical and mental health of preschool children, and this study found that household registration, kindergarten nature, residence environment, and parental exercise support were important influencing factors of daily screen time. Therefore, in the follow-up study, the influence path of parents on preschoolers’ health behavior should be further investigated.

### Limitations

This study was a cross-sectional investigation and cannot draw a causal relationship between variables. Follow-up experimental studies can be used to further reveal the internal factors that affect the physical fitness behavior of preschoolers. Meanwhile, the PA of preschoolers in this study was obtained according to the subjective score, instead of the objective monitoring of their daily PA by accelerometer. Future studies should adopt the method of a subjective score combined with objective monitoring to obtain more accurate PA data.

## Conclusions

This study visually presents the status quo of physical fitness and health behavior of preschool children in southwest China and identifies gender and urban-rural differences in the two variables. On this basis, this study also explores the correlation between preschool children’s physical fitness and healthy behaviors and makes clear the important role of maintaining healthy behaviors (especially MVPA) in improving children’s physical fitness. It was worth noting that this study found that preschool children’s health behaviors were closely related to gender, environment, parents, and other factors, so how to increase the protective factors of children’s health behaviors and controlling the risk factors may be crucial to promoting preschool children to develop good health behaviors.

## Data Availability

No datasets were generated or analysed during the current study.
